# Generalization Enhancement of Visual Reinforcement Learning through Internal States

**DOI:** 10.3390/s24144513

**Published:** 2024-07-12

**Authors:** Hanlin Yang, William Zhu, Xianchao Zhu

**Affiliations:** 1 Institute of Fundamental and Frontier Sciences, University of Electronic Science and Technology of China, Chengdu 611731, China; hanlinyoung@std.uestc.edu.cn (H.Y.); wfzhu@uestc.edu.cn (W.Z.); 2 School of Artificial Intelligence and Big Data, Henan University of Technology, Zhengzhou 450001, China

**Keywords:** visual reinforcement learning, transfer learning, generalization

## Abstract

Visual reinforcement learning is important in various practical applications, such as video games, robotic manipulation, and autonomous navigation. However, a major challenge in visual reinforcement learning is the generalization to unseen environments, that is, how agents manage environments with previously unseen backgrounds. This issue is triggered mainly by the high unpredictability inherent in high-dimensional observation space. To deal with this problem, techniques including domain randomization and data augmentation have been explored; nevertheless, these methods still cannot attain a satisfactory result. This paper proposes a new method named Internal States Simulation Auxiliary (ISSA), which uses internal states to improve generalization in visual reinforcement learning tasks. Our method contains two agents, a teacher agent and a student agent: the teacher agent has the ability to directly access the environment’s internal states and is used to facilitate the student agent’s training; the student agent receives initial guidance from the teacher agent and subsequently continues to learn independently. From another perspective, our method can be divided into two phases, the transfer learning phase and traditional visual reinforcement learning phase. In the first phase, the teacher agent interacts with environments and imparts knowledge to the vision-based student agent. With the guidance of the teacher agent, the student agent is able to discover more effective visual representations that address the high unpredictability of high-dimensional observation space. In the next phase, the student agent autonomously learns from the visual information in the environment, and ultimately, it becomes a vision-based reinforcement learning agent with enhanced generalization. The effectiveness of our method is evaluated using the DMControl Generalization Benchmark and the DrawerWorld with texture distortions. Preliminary results indicate that our method significantly improves generalization ability and performance in complex continuous control tasks.

## 1. Introduction

Visual reinforcement learning plays a crucial role in several practical applications, such as video games [1,2], robotic manipulation [3,4,5,6,7], and autonomous navigation [8,9,10,11]. Compared with reinforcement learning from other data sources, it has gained increasing attention due to its broader range of applications and greater adaptability to diverse environments and devices. This approach allows for overcoming barriers such as unavailability, absence, or corruption of data sources in fields like video games, robotic manipulation, and autonomous navigation. Despite the numerous advantages of this approach and the significant progress achieved, it is still very challenging for vision-based agents to generalize their abilities to unseen environments [12,13,14,15]. This problem, treated as an overfitting problem, is mainly attributed to the high unpredictability inherent in high-dimensional observation space, which severely restricts the deployment of visual reinforcement learning algorithms.

To enhance generalization, both domain randomization (DR) and data augmentation (DA) have been proposed. The underlying assumption behind DR is that the environments’ variation at test time should be effectively covered during training time. When this assumption is valid, the method can obtain relatively good results, but the expansion of the training set and unpredictability of real environments may lead to unstable training, lower sample efficiency, and policies’ suboptimality and high-variance [16]. Furthermore, as the project advances into the actual deployment stage, numerous complex and unanticipated observations can arise. Consequently, it becomes exceedingly difficult to satisfy the initial assumptions. In contrast to DR, DA explicitly manipulates the observation to increase the variability of training data. Recent works have demonstrated better generalization using simple cropping and translation augmentations, but they are still plagued by reduction in sample efficiency and the possibility of divergence. In addition to this, specific methods of DA are found to be task-dependent.

To enhance generalization and maintain sample efficiency bounded in reinforcement learning tasks, in this paper, we propose the Internal States Simulation Auxiliary (ISSA). The learning process of our method is divided into two phases, termed as the transfer learning phase and traditional visual reinforcement learning phase. In the first phase, i.e., the transfer learning phase, two agents, a student agent and a teacher agent, will be trained. The observation of the teacher agent comprises a hybrid integration of visual imagery and internal states, while the student agent relies solely on vision for its observations. The teacher agent has the ability to directly access the environment’s internal states and is used to facilitate the student agent’s training. In the subsequent phase, i.e., the traditional visual reinforcement learning phase, the teacher agent is discarded, and the student agent autonomously learns from visual information in the environments.

**Figure 1:**
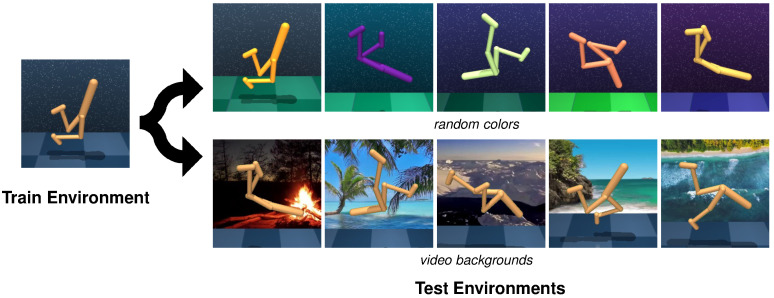
**Illustration of generalization testing on the DMControl-GB.** The model is trained on a fixed background environment and tested on the same environments with a series of unseen backgrounds including *random colors* and *video backgrounds*. *random colors* and *video backgrounds* each encompass easy and hard modes, and the more challenging one, hard, is applied.

**Figure 2:**
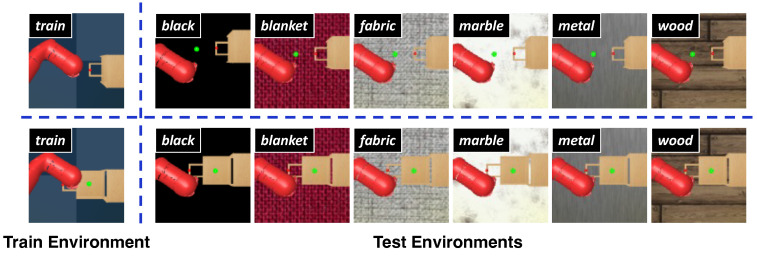
**Illustration of generalization testing on the DrawerWorld with texture distortions.** The upper portion of the diagram represents DrawerOpen, while the lower portion depicts DrawerClose. The model is trained on the fixed *train* texture and evaluated within the same *train* texture as well as in various other environments, including *black*, *blanket*, *fabric*, *metal*, *marble*, and *wood* textures.

To validate the effectiveness of our method, we conducted a series of experiments on the DMControl Generalization Benchmark (DMControl-GB) [17], which is based on continuous control tasks from the DeepMind Control Suite (DMC) [18], and the DrawerWorld with texture distortions [19], which is based on continuous robot control tasks from Meta-World [20]. The first benchmarking platform provides a fixed background setting for model training and different unseen backgrounds including *random colors* and *video backgrounds* for the generalization testing of the model, as shown in Figure 1. Two modes, easy and hard, exist in both *random colors* and *video backgrounds*, and we choose the more challenging settings. The second benchmarking platform, named as DrawerWorld with texture distortions, comprises two tasks: DrawerOpen and DrawerClose. As shown in Figure 2, these tasks involve controlling a robot to open and close a drawer, respectively. The model is trained under the standard grid texture, designated as *train*, and subsequently evaluated within the same *train* texture as well as in various other environments, including *black*, *blanket*, *fabric*, *metal*, *marble*, and *wood* textures.

We select DrQ-v2 [21] to serve as our agents’ base model, and both the student and teacher agents are modified and adapted accordingly. Preliminary results indicate that our method improves generalization ability and sample efficiency. In addition, in some environments, it can significantly improve the performance.

The remainder of this article is organized as follows. Section 2 briefly reviews the related works and preliminaries in visual reinforcement learning, including its generalization and two important methods named DR and DA. Section 3 details the proposed ISSA framework and its details. Section 4 introduces the experiments, including the experimental settings, results, and discussions. Finally, Section 5 draws the conclusion of this study.

## 2. Related Works and Preliminaries

### 2.1. Visual Reinforcement Learning

The framework under traditional reinforcement learning is considered a Markov Decision Process (MDP) [22], formulated by the 5-tuple 〈S,A,r,p,γ〉, where S is the state space, A is the action space, r:S×A→R is a reward function, $p(s_{t+1}\;|\;s_{t},a_{t})$ is the state transition function, and γ∈[0,1) is the discount factor. Beyond that, visual reinforcement learning should be formulated to a Partially Observable Markov Decision Process (POMDP), which can be described as the 6-tuple 〈S,O,A,r,P,γ〉, where O is the high-dimensional observation space (image pixels). The environment evolves according to the internal state $s_{t}\in S$ and returns the high-dimensional observation $o_{t}\in O$, which are, respectively, invisible and visible to the agent. To this end and per common practice [23], three consecutive visual observations $x_{t}=\left\{o_{t},o_{t+1},o_{t+2}\right\}$ are stacked and passed through an image encoder. The output $s_{t}^{\prime }=f_{\xi }\left(x_{t}\right)$ is employed to represent the observations. To avoid ambiguity and enhance readability, the state $s_{t}\in S$ is replaced by an internal state $i_{t}\in I$. And the action $a_{t}$ is obtained by following the policy $\pi_{\phi }\left(\cdot \;|\;s_{t}^{\prime }\right)$, which is parameterized by learnable parameters ϕ. Then, we aim at training the policy $\pi_{\phi }\left(\cdot \;|\;s_{t}^{\prime }\right)$ to maximize the cumulative discounted return $E_{\pi }\left[\sum_{t=1}^{\infty }\gamma^{t}r_{t}|a_{t}∼\pi_{\phi }\left(\cdot \;|\;s_{t}^{\prime }\right),s_{t}^{\prime }∼p\left(\cdot \;|\;i_{t},a_{t}\right),s_{0}^{\prime }∼p\left(\cdot \right)\right]$.

### 2.2. Generalization

In terms of generalization, we consider a set of similar POMDPs, $M=\{M_{1},M_{2},...,M_{n}\}$ that shares the same dynamics and structures. In other words, the remaining five tuples in each of these POMDPs are the same except for observation space, $I_{m}=I_{n},A_{m}=A_{n}$, $r_{m}=r_{n},P_{m}=P_{n},\gamma_{m}=\gamma_{n}\;and\;O_{m}\neq O_{n}\left(1\leq m,n\leq N;m,n\in N\right)$. Ref. [24] formally describes them as ’Block MDPs’. Only one fixed POMDP, denoted as $M_{i}$, is employed for training the policy $\pi_{\phi }\left(\cdot \;|\;s_{t}^{\prime }\right)$. And the generalization ability of the trained policy is defined as the expected cumulative return over other POMDPs contained within the set M.

### 2.3. Domain Randomization

Tobin et al. [3] trained a model on generated fictitious images and, then, transferred it to real images. The assumption of this simple technique is that the real world may appear to the model as just another variation with enough variability in the simulator. Ren et al. [4] focused on how to improve the accuracy of pose estimation based on DR. Specifically, the network is trained on generated images with a high variation in textures and lighting. As mentioned before, the underlying assumption behind DR is that the environments’ variation at test time should be effectively covered during training time. But the expansion of the training set and the unpredictability of real environments may lead to unstable training, lower sample efficiency, and the policies’ suboptimality and high-variance [16].

### 2.4. Data Augmentation

In the context of computer vision, DA has been a prominent way to address the generalization problem by injecting useful priors. It is crucial for the early success of CNNs [25,26] and has promoted semi-supervised and transfer learning [27,28]. Laskin et al. [29] first introduced this technique into reinforcement learning by modifying the replay buffer to be data-augmented. This is a simple plug-and-play method but is very powerful. Since then, DA has been a promising way to address the generalization problem, and a number of algorithms including DrQ [30], SODA [17], SVEA [31], and DrQ-v2 [21] have been developed. DrQ utilized random cropping and regularized Q-functions in conjunction with the off-policy RL algorithm SAC [32]. To address the issues of low sample efficiency and unstable training that emerge from algorithms, which directly learn policies from augmented data, SODA imposes a soft constraint on the encoder that aims to maximize the mutual information between latent representations of augmented and non-augmented data. SVEA incorporates three components to deal with the instability introduced by DA. DrQ-v2 is an enhanced version of DrQ with multiple improvements including shifts in the base algorithm, the addition of multi-step returns, and bilinear interpolation. Despite new challenges such as difficult convergence [31] and the task dependency of specific augmentations introduced by DA, all of the algorithms mentioned above achieved state-of-the-art performance at that time. Therefore, DA remains an essential component of visual reinforcement learning algorithms.

## 3. Method

In this section, we present ISSA, a framework for visual reinforcement learning that leverages internal states to enhance the generalization and sample efficiency of the visual reinforcement learning algorithm. This approach offers a straightforward yet powerful solution to incorporate internal states into the learning process. Additionally, it can seamlessly integrate with various existing algorithms as it does not rely on model-based techniques.

### 3.1. Framework

**Figure 3:**
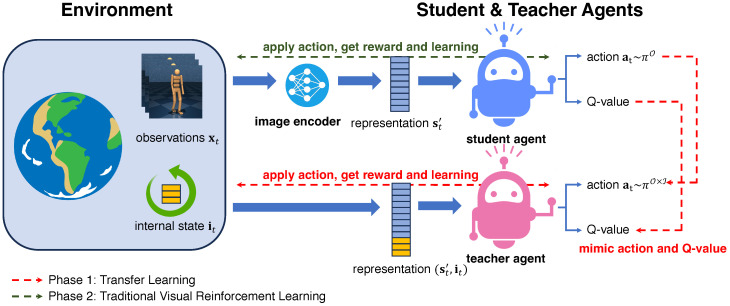
**Schematic overview of ISSA.** The entire process incorporates two-phase learning. Phase 1 is the transfer learning part, indicated by the red line, and it is also the main contribution of this paper. In Phase 1, the teacher agent takes both image representation and internal states as input and makes the action and Q-value estimation based on them. The student agent tries to imitate the teacher’s action and Q-value estimation. In Phase 2, the teacher agent is no longer used, and the student agent learns from the interactions between itself and the environment. The final model, i.e., the student agent trained after Phase 2, is a solely visual reinforcement learning model.

The entire framework is depicted in Figure 3, comprising two distinct learning phases: Phase 1, the transfer learning phase, and Phase 2, the traditional visual reinforcement learning phase. In Phase 1, the teacher agent leverages both the internal state $i_{t}$ and encoded image representation $s_{t}^{\prime }$; after that, the generated action is imitated by the student agent and returned to the environment. In Phase 2, the teacher agent is no longer used; the entire framework becomes an interaction between the environment and the student agent, and the student agent’s policy improves through this process. After the two phases are completed, the final model is the student agent, and it is a visual reinforcement learning model, not a hybrid one.

**Image Encoder.** Applying the pre-trained model from other domains can achieve competitive performance with state-based input and drastically reduce training time and the requirement for advanced hardware. We adopt Resnet-18 [33], pre-trained on ImageNet [34], as our image encoder. The only modification is that the last fully connected layer is removed and replaced with a new learnable layer, which is a routine operation for pre-trained models better adapted to new downstream tasks.

**Agents.** Both the teacher and student agent are based on DrQ-v2, which is a simple actor–critic algorithm for image-based continuous control. DrQ-v2 builds upon DrQ with several changes, including switching to DDPG [35] as the base algorithm, incorporating multi-step return, adding bilinear interpolation to image augmentation, introducing an exploration schedule, and selecting improved hyper-parameters. In the algorithm and image that follow, many of these techniques are not shown due to space constraints.

### 3.2. Transfer Learning Phase Details

In this section, we elaborate on the details of the algorithm and the math behind it. The entire training process, as well as the updating process to the policy and Q-value that occurs in Phase 1, is shown in Algorithm 1.

Lines 2–10 show the training process for Phase 1. In line 4, the teacher agent sample action is based on both internal state and encoded visual observation. The environment continues to evolve, leading to new observations. Both the original and present observations or internal states, in conjunction with the applied action and the resultant reward, are encapsulated and stored within a transition, as delineated in lines 5–7. The policy’s and value function’s update for the teacher agent and student agent take place in lines 8–9. As stated earlier, during Phase 2, the teacher agent is no longer used, and the entire framework degenerates to traditional visual reinforcement learning, specifically DrQ-v2 to the student agent. Consequently, it is unnecessary to delve into the details outlined in line 13.
**Algorithm 1** Full training process for proposed ISSA**Inputs:**$\pi_{\phi^{T}},Q_{\theta_{1}^{T}},Q_{\theta_{2}^{T}},Q_{{\bar{\theta }}_{1}^{T}},Q_{{\bar{\theta }}_{2}^{T}}$: Teacher’s parametric networks for policy and Q functions, both based on combination of visual observation and internal states.$\pi_{\phi^{S}},Q_{\theta_{1}^{S}},Q_{\theta_{2}^{S}},Q_{{\bar{\theta }}_{1}^{S}},Q_{{\bar{\theta }}_{2}^{S}}$: Student’s parametric networks for policy and Q functions, both solely based on visual observation.aug: image augmentation method inherited from DrQ-v2.$f_{\xi },T_{s},T_{r},B,\alpha ,\tau$: parametric network for image encoder, training steps for transfer learning and reinforcement learning, mini-batch size, learning rate and target update rate.1:**procedure** Main2:    $s_{0}^{\prime }\leftarrow f_{\xi }\left(x_{0}\right)$                 ▹ Get encoded state for initial observations3:    **for** *t*:=0 **to** $T_{s}$ **do**                 ▹ Transfer learning part4:        $a_{t}∼\pi_{\phi^{T}}\left(\cdot \;|\;s_{t}^{\prime },i_{t}\right)$                 ▹ Sample action based on teacher’s policy5:        $x_{t+1}\leftarrow p\left(\cdot \;|\;i_{t},a_{t}\right)$                 ▹ Get consecutive observations6:        $s_{t+1}^{\prime }\leftarrow f_{\xi }\left(x_{t+1}\right)$                 ▹ Get encoded state for timestep t+17:        $D\leftarrow D\;\cup \;(x_{t},i_{t},a_{t},r\left(i_{t},a_{t}\right),x_{t+1},i_{t+1})$                 ▹ Store transition8:        UpdateCriticTansfer(D)                 ▹ Update critic for teacher and student9:        UpdateActorTransfer(D)                 ▹ Update policy for teacher and student10:    **end for**                 ▹ End of Phase 1 (transfer learning phase)11:    $s_{0}^{\prime }\leftarrow f_{\xi }\left(x_{0}\right)$                 ▹ Get encoded state for initial observations12:    **for** *t*:=0 **to** $T_{r}$ **do**                 ▹ Reinforcement learning part13:        Traditional reinforcement learning based on $\pi_{\phi^{S}},Q_{\theta_{1}^{S}},Q_{\theta_{2}^{S}},Q_{{\bar{\theta }}_{1}^{S}},Q_{{\bar{\theta }}_{2}^{S}}$14:    **end for**                 ▹ End of the entire training process15:**end procedure**16:**procedure** UpdateCriticTransfer(D)17:    $\{\left(x_{t},i_{t},a_{t},r_{t:t+n-1},x_{t+n},i_{t+n}\right)\}∼D$                 ▹ Sample a mini batch of *B* transitions18:    $s_{t}^{\prime },s_{t+n}^{\prime }\leftarrow f_{\xi }\left(aug\left(x_{t}\right)\right),f_{\xi }\left(aug\left(x_{t+n}\right)\right)$                 ▹ Data augmentation and encoding19:    $a_{t+n}∼\pi_{\phi^{T}}\left(\cdot \;|\;s_{t+n}^{\prime },i_{t+n}\right)$                 ▹ Sample action for timestep t+n20:    Compute $L_{\theta_{1}^{T},\xi },L_{\theta_{2}^{T},\xi },L_{\theta_{1}^{S},\xi },L_{\theta_{2}^{S},\xi }$ using Equation (1).21:    $\xi \leftarrow \xi -\alpha \nabla_{\xi }\left(L_{\theta_{1}^{T},\xi }+L_{\theta_{2}^{T},\xi }+L_{\theta_{1}^{S},\xi }+L_{\theta_{2}^{S},\xi }\right)$                 ▹ Update encoder22:    $\theta_{k}^{i}\leftarrow \theta_{k}^{i}-\alpha \nabla_{\theta_{k}^{i}}L_{\theta_{k}^{i},\xi }\;\;k\in \left\{1,2\right\},i\in \left\{T,S\right\}$                 ▹ Update Q functions23:    ${\bar{\theta }}_{k}^{i}\leftarrow \left(1-\tau \right){\bar{\theta }}_{k}^{i}+\tau \theta_{k}^{i}\;\;k\in \left\{1,2\right\},i\in \left\{T,S\right\}$                 ▹ Soft update target-Q functions24:**end procedure**25:**procedure** UpdateActorTransfer(D)26:    $\{\left(x_{t},i_{t}\right)\}∼D$                 ▹ Sample a mini batch of *B* observations and internal states27:    $s_{t}^{\prime }\leftarrow f_{\xi }\left(aug\left(x_{t}\right)\right)$                 ▹ Data augmentation and encoding28:    $a_{t}∼\pi_{\phi^{T}}\left(\cdot \;|\;s_{t}^{\prime },i_{t}\right)$                 ▹ Sample action for timestep t+n29:    Compute $L_{\phi^{T}},L_{\phi^{S}}$ using Equations (3) and (4).30:    $\phi^{i}\leftarrow \phi^{i}-\alpha \nabla_{\phi^{i}}L_{\phi^{i}}\;\;i\in \left\{T,S\right\}$                 ▹ Update teacher’s and student’s policy31:**end procedure**

Since both agents are inherited from DrQ-v2, which adopts DDPG coupling with Double Q-learning, each agent is equipped with a pair of parameterized networks $Q_{\theta_{1}},Q_{\theta_{2}}$ designated to estimate Q-values, as well as two target networks $Q_{{\bar{\theta }}_{1}},Q_{{\bar{\theta }}_{2}}$ aimed at reducing the overestimation bias of target Q-values. A mini-batch of transitions $\tau =\{\left(x_{t},i_{t},a_{t},r_{t:t+n-1},x_{t+n},i_{t+n}\right)\}$ is sampled from the replay buffer D. The observations $x_{t}$ and $x_{t+n}$ are then augmented and encoded. The loss function of the critic networks for both the teacher agent and student agent (for student agent, parameter $i_{t}$ is ignored) is as follows:

(1) $$L_{\theta ,\xi }=E_{\tau ∼D}\left[{\left(Q_{\theta }\left(s_{t}^{\prime },i_{t},a_{t}\right)-y\right)}^{2}\right],$$

with an n-step TD target *y*:

(2) $$y=\sum_{i=0}^{n-1}\gamma^{i}r_{t+i}+\gamma^{n}\min_{k=1,2}Q_{{\bar{\theta }}_{k}^{T}}\left(s_{t}^{\prime },i_{t},a_{t}\right).$$

The policy network, or actor, for the teacher agent shares the same update process with DrQ-v2. It is trained using DPG with the following loss:

(3) $$L_{\phi^{T}}=-E_{x_{t}∼D}\left[\min_{k=1,2}Q_{\theta_{k}^{T}}\left(s_{t}^{\prime },i_{t},a_{t}\right)\right],$$

where $s_{t}^{\prime }=f_{\xi }\left(aug\left(x_{t}\right)\right)$, and $a_{t}$ is sampled from $\pi_{\phi^{T}}\left(\cdot \;|\;s_{t}^{\prime },i_{t}\right)$. And to train the student agent to imitate the teacher agent’s behavior, we use the following loss function:

(4) $$\begin{array}{cc} L_{\phi^{S}} & =D_{KL}(\pi_{\phi^{T}}\left(\cdot \;|\;s_{t}^{\prime },i_{t}\right)\left|\right|\pi_{\phi^{S}}\left(\cdot \;|\;s_{t}^{\prime }\right))\\ \; & =\int_{-\infty }^{\infty }\pi_{\phi^{T}}\left(x\;|\;s_{t}^{\prime },i_{t}\right)\ln\frac{\pi_{\phi^{T}}\left(x\;|\;s_{t}^{\prime },i_{t}\right)}{\pi_{\phi^{S}}\left(x\;|\;s_{t}^{\prime }\right))}dx \end{array}$$

where $D_{KL}$ stands for Kullback–Leibler divergence.

## 4. Experiments

This section presents the empirical evaluation results of our proposed method on an extensive set of visual reinforcement learning tasks from the DMControl-GB and DrawerWorld with texture distortions. We provide comparisons to previous methods, both model-free and model-based methods.

### 4.1. Setups

During the first training phase, the student agent is trained for 50 k steps along with the teacher agent in a manner that the teacher agent interacts directly with the environment, and the student agent imitates the teacher agent’s action. Upon that, the student agent is trained for another 50 k steps. Each action will repeat 3 times. We employ the pre-trained ResNet18 model as the encoder. To be more specific, the last fully connected layer of the model is discarded and replaced with an untrained and trainable fully connected layer to better accommodate the task-specific learning requirements. In the architecture of the teacher agent, the embedding of observations, i.e., output of the encoder, will be concatenated with the internal state vector prior to being taken as the input of the policy network and the Q-value networks.

### 4.2. Evaluation on Generalization Ability

To validate the generalization ability of the ISSA on the DMControl-GB, we chose multiple renowned algorithms as baselines. These include the following: SAC, a weak baseline yet still prevalent off-policy traditional RL algorithm; DrQ, which combines traditional DQN with data-regularized Q, specifically for processing visual stimuli; SODA, where policy learning is decoupled from DA techniques; SVEA, a former state-of-the-art visual reinforcement learning algorithm that reduces high variance in Q-targets; and DrQ-v2, an enhanced version of DrQ that introduces multiple improvements to stabilize policy learning and speed up computation.

Table 1 categorizes the results with *random colors* presented above and *video backgrounds* listed below. The best and second-best results in each environment are highlighted in bold and underlined, respectively. As shown in the table, our method achieves superior outcomes in 8 out of the 10 settings, with an average improvement of 6.8% compared to the second-best. Although this improvement may seem modest, it is important to consider that, in most cases, the comparison is with SVEA, which remains the state-of-the-art algorithm and achieves the vast majority of the second-best results.

In order to further validate the generalization abilities of our proposed ISSA method, we conducted generalization benchmarking on the DrawerWorld with texture distortions. We used four algorithms, SAC, DrQ, PAD [36], and SVEA, as baselines. PAD represented the preceding state-of-the-art (SOTA) method, encompassing techniques such as unsupervised policy adaption and auxiliary task prediction during test time to optimize the visual encoder. Table 2 categorizes the results of DrawerOpen and DrawerClose tasks within all textured environments including *train*, *black*, *blanket*, *fabric*, *metal*, *marble*, and *wood*. The experimental results demonstrate that our method consistently surpassed all the baselines under all conditions. Furthermore, our maximum improvement reached up to 60%. And we achieved double-digit enhancements in 9 out of the 14 settings.

### 4.3. Evaluation on Sample Efficiency

**Figure 4:**
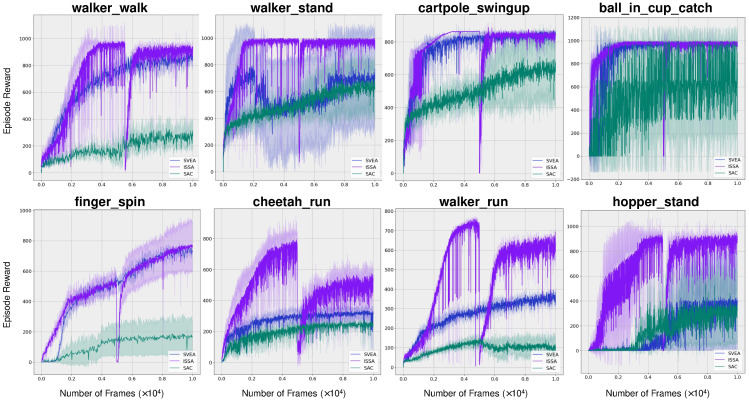
**Training sample efficiency.** Average episode rewards on eight challenging tasks. And the shaded area is std. deviation. Our proposed method ISSA either matches or surpasses the sample efficiency and asymptotic performance of both SVEA and SAC in all evaluated tasks. Under walker_walk, walker_stand, walker_run, chetaah_run, and hopper_stand, ISSA exhibits significantly greater sample efficiency and final outcomes. Additionally, in walker_stand, cheetah_run, walker_run, and hopper_stand, the final outcome improvements are more than 50%.

The sample efficiency of ISSA was assessed across various tasks derived from the DMControl-GB. As illustrated in Figure 4, our proposed method, ISSA, demonstrates superior sample efficiency during the initial transfer learning phase. Then, a significant but briefly slump occurs upon transitioning to the subsequent traditional visual reinforcement learning phase, which is aimed at developing a solely vision-based reinforcement agent. Despite this, the performance quickly improves and eventually matches the initial level. Figure 4 indicates our proposed method either matches or surpasses the sample efficiency and asymptotic performance of both SVEA and SAC in all evaluated tasks. And under relatively demanding tasks walker_walk, walker_stand, walker_run, chetaah_run, and hopper_stand, our algorithm not only exhibits significantly greater sample efficiency but also substantially enhances the final outcomes. Additionally, it can be seen from the diagram, in environments walker_stand, cheetah_run, walker_run, and hopper_stand, we achieved more than 50% improvements.

### 4.4. Abalation Study

**Figure 5:**
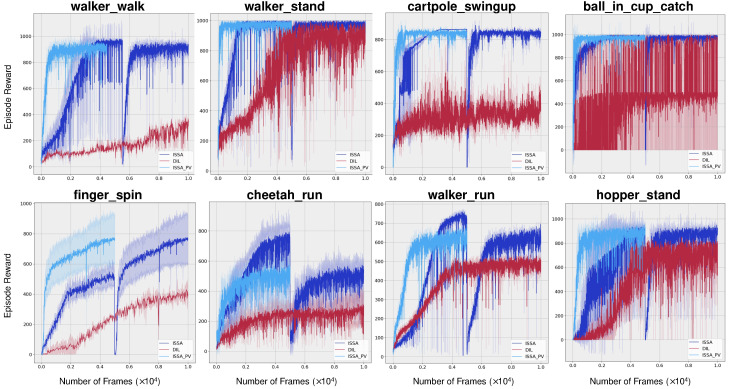
**Validation of knowledge transfer.** DIL denotes the student model learned directly from environments without a transfer learning phase. ISSA_PV denotes the curve portion of the traditional reinforcement learning phase, which is extracted from ISSA directly. This experiment aims to verify whether the knowledge has been successfully imparted to the student model. The results clearly demonstrate the pronounced superiority of ISSA_PV over ISSA and DIL, indicating that the knowledge was effectively transferred to the student agent.

This section aims to validate the significance of the transfer learning phase and ascertain that the experiences acquired by the teacher model are successfully transferred to the student model. We use the same eight challenging environments in the evaluation of training sample efficiency. To validate that the experiences acquired by the teacher model are effectively transferred to the student model, we firstly train a student model without a transfer learning phase, denoted as DIL. Then, we extract the curve portion of the traditional reinforcement learning phase—specifically, the stage where the final agent relies solely on vision—and placed it at the forefront, denoted by ISSA_PV. The results are shown in Figure 5. It is evident that ISSA_PV holds a pronounced superiority in comparison to ISSA and DIL. Consequently, the implementation of the transfer learning phase substantially enhances the performance of the final agent, and the experiences acquired by the teacher model are effectively transferred to the student model. It not only surpasses the teacher model, which has direct access to environment’s internal states, but it also outperforms the student model trained from scratch.

## 5. Conclusions

In this study, we proposed the ISSA method, leveraging internal states to enhance the generalization of visual reinforcement learning algorithms. Our method contains two agents, a teacher agent and a student agent: the teacher agent has the ability to directly access the environment’s internal states and is used to facilitate the student agent’s training; the student agent receives initial guidance from the teacher agent and subsequently continues to learn independently. From the perspective of how to train the model, our method can be divided into two phases, the transfer learning phase and traditional visual reinforcement learning phase.

To validate our method, we chose DMControl-GB and DrawerWorld with texture distortions as benchmarking platforms. Experiments demonstrated that our method significantly improved both generalization and sample efficiency in previously unseen environments’ backgrounds. Additionally, to validate whether the knowledge has been successfully imparted to the student model, we conducted an ablation study and chose ISSA_PV and DIL as baselines. The results clearly show that the knowledge was successfully transferred to the student model.

Although we chose DrQ-v2 as our base model, our method was model-agnostic. Thus, our method may be a promising approach for other reinforcement learning experiments and may be expected to play an increasingly vital role in future research. We believe that further research can continue to explore how to more reasonably and efficiently utilize the models and knowledge obtained in the transfer learning phase, for example, whether the knowledge obtained in the first phase can be directly used by the student agent in the second phase, rather than obtaining training data by interacting with a new environment. Our future work will focus on this area.

## Figures and Tables

**Table 1:** **Generalization ability comparison on the DMControl-GB**. The test performance (episode return) of methods trained in a fixed environment and evaluated on the *random colors* and *video backgrounds* benchmark from the DMControl-GB. We report mean and std. deviation of 5 runs. The optimal and suboptimal outcomes are, respectively, highlighted in bold and underlined. ISSA achieves superior outcomes in 8 out of 10 settings.

DMControl-GB (*random colors*)	SAC [32]	DrQ [30]	SODA [17]	SVEA [31]	DrQ-v2 [21]	ISSA (Student Model)
walker_walk	173 ± 23	520 ± 91	660 ± 59	760 ± 145	673 ± 43	**837 ± 105 (+10.1%)**
walker_stand	371 ± 90	770 ± 73	930 ± 12	942 ± 26	861 ± 24	**978 ± 85 (+3.8%)**
cartpole_swingup	248 ± 24	586 ± 52	819 ± 22	835 ± 20	814 ± 80	**873 ± 28 (+4.6%)**
ball_in_cup_catch	172 ± 21	365 ± 210	949 ± 19	961 ± 7	469 ± 99	**997 ± 23 (+3.7%)**
finger_spin	372 ± 64	776 ± 134	896 ± 82	**977 ± 5**	730 ± 110	970 ± 19
**DMControl-GB (** ***video backgrounds*** **)**	**SAC [32]**	**DrQ [30]**	**SODA [17]**	**SVEA [31]**	**DrQ-v2 [21]**	**ISSA (student model)**
walker_walk	142 ± 17	516 ± 93	692 ± 68	819 ± 71	719 ± 27	**894 ± 27 (+9.1%)**
walker_stand	349 ± 103	790 ± 76	893 ± 12	**961 ± 8**	673 ± 19	935 ± 23
cartpole_swingup	248 ± 23	579 ± 47	758 ± 62	782 ± 27	267 ± 41	**836 ± 76 (+6.9%)**
ball_in_cup_catch	151 ± 36	365 ± 210	875 ± 56	871 ± 106	469 ± 99	**967 ± 57 (+10.5%)**
finger_spin	280 ± 17	776 ± 134	793 ± 128	803 ± 33	780 ± 72	**848 ± 24 (+5.6%)**

**Table 2:** **Generalization ability comparison on the DrawerWorld**. The primary objective of these experiments is to assess the model’s generalization abilities across various textured backgrounds. Specifically, the model is trained under the *train* textured background and subsequently evaluated in all textured environments including *train* itself. Success rate (%) is adopted as the criterion for evaluation. The experimental results demonstrate that our method consistently surpasses baselines under all tested conditions. We report mean and std. deviation of 5 runs. The optimal and suboptimal outcomes are, respectively, highlighted in bold and underlined.

Task (Success %)	Setting	SAC [32]	DrQ [30]	PAD [36]	SVEA [31]	ISSA (Student Model)
	*train*	98 ± 2	**100 ± 0**	84 ± 7	87 ± 5	**100 ± 0 (+0)**
	*black*	95 ± 2	99 ± 1	95 ± 3	56 ± 29	**100 ± 0 (+1)**
	*blanket*	28 ± 8	28 ± 15	54 ± 6	35 ± 23	**91 ± 4 (+37)**
DrawerOpen	*fabric*	2 ± 1	0 ± 0	20 ± 6	25 ± 12	**85 ± 3 (+60)**
	*metal*	35 ± 7	35 ± 35	81 ± 3	83 ± 10	**98 ± 1 (+15)**
	*marble*	3 ± 1	0 ± 0	3 ± 1	18 ± 24	**56 ± 8 (+38)**
	*wood*	18 ± 5	5 ± 3	39 ± 9	40 ± 6	**93 ± 4 (+53)**
	*train*	**100 ± 0**	91 ± 7	95 ± 3	60 ± 23	**100 ± 0 (+0)**
	*black*	75 ± 4	65 ± 13	64 ± 9	55 ± 16	**100 ± 0 (+25)**
	*blanket*	0 ± 0	44 ± 23	0 ± 0	4 ± 6	**83 ± 8 (+39)**
DrawerClose	*fabric*	0 ± 0	80 ± 14	0 ± 0	3 ± 4	**87 ± 3 (+7)**
	*metal*	0 ± 0	51 ± 25	2 ± 2	47 ± 11	**97 ± 4 (+46)**
	*marble*	0 ± 0	86 ± 10	0 ± 0	25 ± 3	**89 ± 2 (+3)**
	*wood*	0 ± 0	50 ± 32	12 ± 2	26 ± 2	**70 ± 7 (+20)**

## Data Availability

The data presented in this study are available on request from the corresponding author due to the need for further research.

## Abbreviations

The following abbreviations are used in this manuscript:

DR	Domain Randomization
DA	Data Augmentation
DMControl-GB	DMControl Generalization Benchmark
DMC	DeepMind Control Suite
MDP	Markov Decision Process
POMDP	Partially Observable Markov Decision Process
